# Tax Incentives, Tax Enforcement, and Enterprise R&D Investment: Evidence From Chinese A-Share Listed

**DOI:** 10.3389/fpsyg.2022.953313

**Published:** 2022-07-08

**Authors:** Ying Sun

**Affiliations:** Department of Economics and Management, Shanghai Administration Institute, Shanghai, China

**Keywords:** tax incentives, tax enforcement, policy effect, R&D investment, the nature of property rights

## Abstract

This study investigates the relationship between tax incentives, tax enforcement, and R&D investment in Chinese enterprises. Tax compliance is an important part of organizational behavioral psychology, which will impact organizational innovation activities. Therefore, the present study utilizes the panel data of Chinese A-share listed companies collected from the China Stock Market & Accounting Research (CSMAR) database from 2011 to 2020 to conduct empirical research. It tests the policy effect of tax incentives on enterprise R&D investment and examines the impact of tax enforcement on enterprise R&D behavior and its moderating effect on the relationship between tax incentives and enterprise R&D investment. The results show that China’s preferential tax policies positively affect enterprise innovation activities, and stable and continuous tax incentives can stimulate enterprises to increase R&D investment. Increasing the intensity of tax enforcement has a significant positive impact on enterprise R&D investment, which means that the promotion effect of “governance effect” and “incentive effect” caused by tax enforcement may exceed the negative impact brought by its “taxation effect” and “rent-seeking effect.” The study suggests that with the strengthening of tax enforcement, the promotion effect of tax incentives is weakened, which is only reflected in non-state-owned enterprises. Finally, we propose relevant policy recommendations based on the research results. This includes further optimizing the collocation of tax types and preferential methods, encouraging enterprises to face up to the role of tax enforcement as an external governance mechanism, and promoting the tax department to standardize the enforcement process and taxation services. The paper presents a range of theoretical and practical implications for both firm managers and policymakers.

## Introduction

The world is experiencing profound shifts unseen in a century, and the global spread of the COVID-19 pandemic has accelerated the evolution of these shifts. A wider and deeper new scientific and technological revolution and industrial transformation are booming on a global scale. Several key cutting-edge technologies show the trend of multiple breakthroughs, cross-convergence, and group leaps forward. At the same time, these will also be accompanied by the emergence of many new business forms, production methods, and business models. Scientific and technological innovation has become a key variable affecting and changing the global economic landscape. According to the data from *The Global Innovation Index* (GII), China’s ranking has risen sharply in recent years, jumping from 34th in 2012 to the 12th in 2021. It is the only middle-income economy in the world’s top 30. Although China has become an important contributor to global scientific and technological innovation, there is still a big gap between China’s and the world’s advanced level, and the situation that others control key core technologies has not been fundamentally changed. The “14th Five-Year Plan” clearly calls for “boosting the main role of enterprises in innovation” and states “more efforts will be made to implement inclusive policies such as granting an extra tax deduction on R&D costs and offering preferential tax treatment to high-tech enterprises.” Tax policy has always been a key tool for stabilizing economic growth, promoting industrial transformation and upgrading, and encouraging balanced regional development. Because of its precise force and outstanding structural characteristics, the use of tax incentives to stimulate corporate innovation has become a mainstream trend worldwide.

The policy effect of tax incentives on enterprise innovation has been a long-term research focus of scholars. Academia has carried out many empirical tests at different levels on the overall effect of the policy, and the research results are diverse. The price spillover and knowledge spillover brought by technology investment mean enterprise innovation has strong externality characteristics, which leads to a lower willingness toward enterprise R&D investment and technological innovation (Shah et al., 2019; Sarfraz et al., 2020). Most research results demonstrate that tax incentives can effectively avoid the risks and uncertainties caused by the above situation and play an important role in promoting private R&D investment and innovation activities (Rao, 2016; Dai and Chapman, 2022). The long-term incentive effect of policies is generally better than that of the short-term (Guellec and Pottelsberghe, 2003; Mckenzie and Sershun, 2010). However, some scholars hold the opposite view, arguing that the policy cost of tax incentives is relatively high, and they are inefficient in solving the problem of market failure caused by innovation externalities. In addition, the incentive goal of tax preference is not necessarily consistent with maximizing the company’s interests. Incentive distortion may occur in the implementation of the policy, and there are cases where companies invest the saved funds for purposes other than R&D activities, resulting in a deviation from the original policy goals (Liu, 2016; Liu and Wang, 2020), and as such, the incentive effect on enterprise innovation investment is minimal (Thomson, 2010). Tax preference does not significantly improve the willingness of enterprises to carry out breakthrough innovation, nor the degree of recognition by the technology market (Han and Chen, 2021). The marginal impact of tax incentives on enterprise innovation is uncertain and the effect of policy implementation depends on the number of incentives that enterprises enjoy. Still, a simple increase in incentive intensity does not necessarily improve the innovation capability of enterprises (Lin et al., 2013). Does the debate over policy effects imply that there may be other complex influences between tax incentives and corporate R&D activities? Existing works in the literature take the preferential tax rate as the measurement index and provide rich research on the relationship between tax incentives and R&D investment from the policy design perspective. Still, the tax enforcement behavior of the tax department in the policy implementation process is also a key factor influencing the effect of tax policy tools. Given this, this paper uses the data of Chinese A-share listed companies from 2011 to 2020 as a research sample to empirically test the policy effect of tax incentives on enterprise R&D investment and examine the impact of tax enforcement on enterprise R&D behavior and the moderating effect of tax enforcement in the relationship between tax incentives and enterprise R&D investment. In this way, it responds to the debate on the economic effects of tax policies on micro-subjects and also adds practical evidence from China to explain the effects of preferential tax policies fully.

The main research contributions of this paper are: (1) A focus on the synergistic effect of tax enforcement on tax incentives and the provision of a new perspective for the study of tax incentives and enterprise innovation; and (2) Expansion of the relevant research on tax enforcement. Regarding the first point, most of the existing literature focuses on evaluating the effectiveness of tax incentives from the policy design perspective. Few studies consider the possible impact of tax enforcement behavior on the effect of policy implementation. This paper incorporates the factors of policy implementation into the research framework of tax policy and corporate innovation activities. It examines the synergistic effect of the two and the heterogeneity caused by differences in internal characteristics of enterprises. To a certain extent, this paper provides a new perspective for evaluating the effect of fiscal and taxation policies. In terms of the second key contribution, the existing literature mostly discusses the impact of tax enforcement on enterprise earnings management (Sun et al., 2019; Li et al., 2021), financing constraints (Cai et al., 2021), tax burden level (Li et al., 2020), enterprise tax avoidance (Tang et al., 2021), and stock price crash risks (Jiang, 2013). From the perspective of R&D activities, this paper offers a new interpretation path for the role of tax enforcement, provides new empirical evidence for evaluating the impact of the government’s strengthening of tax enforcement, and deepens the understanding of the role of tax enforcement in enterprise innovation.

## Theoretical Analysis and Research Hypotheses

### Tax Incentives and Enterprise Innovation

Preferential tax policies are the direct transfer of part of the economic interests of the micro-market entities by the government. As an effective tool for supporting innovation in a market-oriented way, they can actively play a role in the government’s feedback on R&D and innovation activities, and affect the marginal value of enterprise innovation behavior. First, preferential tax policies have a “cost-effect” on innovation investment, which encourages enterprises to strengthen the allocation of R&D resources. The characteristics of the long investment cycle and highly uncertain income of innovation activities mean that enterprises need to have a strong financial position to engage in them. In addition, the current intellectual property protection system is not perfect, and the innovation achievements of enterprises are easily replaced by imitation, which will further reduce their expected income from R&D investment, resulting in a lack of willingness and motivation to conduct independent R&D. An indirect tax preference for innovative activities can reduce the marginal cost of enterprise R&D, and direct tax preference given to corporate entities can enable investment in high-complexity and high-risk R&D behaviors to obtain certain risk compensation (Ma et al., 2017). The internal resource allocation of an enterprise will be affected by the level of the tax burden. Tax incentives directly make up for part of the R&D capital expenditure of the enterprise. By reducing the investment cost and investment risk in the innovation process, and improving the level of innovation income, the impact of the externalities of innovation activities can be smoothed as much as possible to balance cost and income and encourage enterprises to increase R&D investment (Shehzad et al., 2020; Tian et al., 2020).

Second, preferential tax policies can effectively alleviate the pressure of endogenous financing constraints and increase the enthusiasm of innovation entities. The uncertainties and risks inherent in innovation activities increase the difficulty for enterprises to obtain external market funds. Information asymmetry and lack of collateral further exacerbate the financial frictions and financing constraints faced by enterprise R&D activities (Brown and Petersen, 2010). To reduce the capital cost of innovation and its high adjustment cost, and consider the information protection of innovation achievements or proprietary technology, companies usually choose sustainable endogenous financing as the main source of funding for innovation activities. Enjoying preferential tax policies can reduce the tax payment of enterprises, plus increase the company’s retained earnings and operating cash flow, thereby indirectly enhancing the supply of funds for innovation entities, effectively alleviating the pressure of capital shortages for enterprises with high financing constraints, and improving the level of investment in R&D activities and innovation efficiency.

Third, preferential tax policies have the function of a “wind vane,” which can send positive signals to the inside and outside of the enterprise. On the one hand, tax incentives reflect the government’s will to support scientific and technological innovation, release a positive signal to enterprises to guide and encourage their innovation, improve decision-maker expectations for future cash flow increases, and to a certain extent stimulate enterprises’ comprehensive innovation behavior (Wang and Li, 2017). On the other hand, tax incentives can be regarded as an invisible “label” recognized by the government, which is beneficial for enterprises to attract potential customers and enhance the market effect of innovative products, thus prompting enterprises to invest more resources in innovation activities (Kleer, 2010). Fourth, tax incentives have the characteristics of wide coverage and strong fairness, leaving enterprises with more room for independent decision-making, which can reduce the distortion of government intervention (Liu and Wang, 2020). Tax incentives can reduce the administrative burden, reduce the risk of the government “picking losers” or “system arbitrage” by enterprises, while enjoying preferential policies may reduce the motivation of enterprises to seek rent and the cost of tax avoidance, and use resources for R&D and innovation (Cai et al., 2021). Hence, we propose the following hypothesis:

*H1(a):* Enterprises enjoying tax incentives can promote their R&D investment.

Adverse selection, moral hazard, and incentive dislocation that preferential tax policies may cause will harm the policy effect, especially when the tax incentive intensity is large (Bloom et al., 2002; Liu, 2016). Firstly, the complexity of economic activities and the high cost of obtaining information determine that the government cannot fully grasp the information chain of market players when designing specific tax preferential policies. The huge differences between various industries have exacerbated the “scarcity” of effective information and the difficulty of decision-making. The government can only adopt simplified “one-size-fits-all” incentive policies for specific industries or around a certain policy goal, which limits the choice of policies for enterprises. At the same time, the objective conditions of information asymmetry will enhance the possibility of the “adverse selection” of market entities. Benefiting from the advantages of information acquisition, enterprises can adjust their strategies according to their needs and preferences to meet policy standards or seek tax arbitrage and tax evasion through deliberate concealment, whitewashing business performance, and false declarations to reduce the effective tax rate. Research has confirmed that tax incentives could stimulate companies to conduct R&D manipulation to a certain extent by increasing their reported R&D investment to take advantage of the super deduction policy or the high-tech enterprise tax reduction policy to reduce their tax burden. Although companies report higher R&D investment, it may not effectively improve their innovation ability and R&D performance (Wu et al., 2013). Tax incentives are less likely to stimulate risky research projects with potentially high social and long-term economic return rates, instead being more likely to induce firms to prefer development activities over research activities (Dai et al., 2020). The false innovation signals released by enterprises eventually lead to the deviation and distortion of the effect of tax incentives.

Secondly, in the market structure with asymmetric information, since the policy intention of government tax incentives is not necessarily consistent with maximizing the interests of market players, enterprises have the motivation to invest the policy resources and save funds for other uses other than R&D and innovation activities, thus creating a “moral hazard” problem. Strong concealment and loose budget constraints lead to preferential tax management practices that are more likely to breed rent-seeking opportunities and increase market distortions (Gao and Mao, 2013). Some tax incentives set “threshold-type” identification standards for market players, enhancing enterprises’ institutional environment (Li et al., 2016). The opportunistic behavior of enterprises and the possibility of “government-enterprise collusion” will further hinder the realization of policy goals.

Thirdly, the lack of restraint mechanisms in China’s tax incentives is an alternative strategy to alleviate information asymmetry, which causes the rights and interests enjoyed by enterprises to be misaligned with their responsibilities (Liu, 2016). Incentive dislocation will exacerbate adverse selection and the moral hazard of market players. Finally, reducing the tax burden may not necessarily promote innovation levels in the long run (Herbig et al., 1994). Tax incentives transfer part of economic benefits and reduce government revenue, which may reduce government spending on education, infrastructure, research, and other social public goods and services (Cai et al., 2021), which is not conducive to creating a good innovation environment. The above factors lead to the unsatisfactory implementation effect of tax incentives in some industries or enterprises and even have a restraining effect that is contrary to the original intention of the policy. Hence, we propose the following hypothesis:

*H1(b):* Enterprises that enjoy tax incentives can inhibit their R&D investment.

### The Impact of Tax Enforcement on the Effect of Tax Incentives

Tax enforcement is a way for the government to exercise public rights, and it is an important external factor that can affect the operation of enterprises and their economic consequences. The interaction of the “taxation effect,” “rent-seeking effect,” “governance effect,” and “incentive effect” of tax enforcement makes its impact on the policy effect of tax incentives and enterprise innovation more complicated.

Regarding the “taxation effect,” taxation is the government’s “mandatory” sharing of business income. Its rigid constraints on time and amount also make it more restrictive on the internal cash flow of enterprises than general debts. The improvement of tax enforcement will directly increase the actual tax burden of enterprises, thereby crowding out retained earnings and cash flow and exacerbating the possibility of financing constraints (Yu et al., 2015). At the same time, subject to limited financing channels and high financing costs, enterprises will use tax avoidance to supplement endogenous financing. The tax avoidance motive becomes stronger as the degree of financing constraints increases (Edwards et al., 2012). However, with the application of modern information technologies such as big data and cloud computing, the tax department’s ability to supervise tax-related information of enterprises has been greatly improved, resulting in a significant increase in the cost of tax evasion. Deterred by stricter supervision due to the illegal entry into the “blacklist” (Sun et al., 2019), it is more difficult for enterprises to use sophisticated tax avoidance techniques to reduce cash outflows. The strengthening of tax enforcement compresses the tax avoidance space, weakens the internal financing capacity, and thus reduces the funds invested in R&D and innovation activities.

In terms of the “rent-seeking effect,” China’s current tax system has reserved a huge “enforcement space” in its initial design (Gao, 2006), and the existence of tax avoidance space for some taxes makes the amount of tax paid dependent on the strength of enforcement. At the same time, tax authorities have discretionary power in tax enforcement. The selectivity and flexibility of the implementation process makes it a “negotiated” enforcement to a certain extent, and the scale and depth of enforcement are more susceptible to human interference (Zhao et al., 2019). In addition to explicit taxes and fees, enterprises may also need to bear the administrative burden caused by informal activity expenditures, such as a series of hidden expenses such as fines, apportionments, and corruption costs from tax authorities, and the high time cost and transaction cost caused by tax inspections, interviews and on-site verification and control deductions, which will enhance the actual tax burden perceived by enterprises. It also induces enterprise managers to spend limited resources and energy on “rent-seeking” activities with higher returns to reduce the intensity of tax enforcement. Therefore, the stronger the tax enforcement, the more serious the rent-seeking behavior that enterprises may take, and the increase of implicit non-productive expenditure will inhibit R&D investment. Hence, we propose the following hypothesis:

*H2(a):* The increase in tax enforcement intensity harms enterprise R&D investment.

Regarding the “governance effect,” the government can be regarded as a “special shareholder” of the enterprise from the perspective that the government can compulsorily share corporate profits through taxation (Desai et al., 2007). Tax enforcement is an effective external governance mechanism, especially the big data tax enforcement represented by “Golden Tax Phase III,” which further improves the information transparency of data sources and the ability to analyze enterprise data, moving the dynamic supervision of the government forward (Sun et al., 2019), which will significantly increase the income tax cost of upward earnings management of enterprises and restrict the degree of aggressive taxation. At the same time, increasing the intensity of tax enforcement can reduce “tunneling behaviors” such as the capital occupation of controlling shareholders and related transactions (Zeng and Zhang, 2009), and inhibit opportunistic behaviors such as management’s internal transactions and on-the-job consumption, and reduce their self-interest. In this way, the principal-agent conflict between major shareholders and minority shareholders and between shareholders and management can be effectively alleviated (Desai et al., 2007), and the consistency of their value maximization goals can be enhanced (Li et al., 2017), which urge managers to invest resources in more valuable productive R&D and innovation projects, and provide good sustainable resource support for innovation activities (Sun et al., 2019; Lan et al., 2021).

In terms of the “incentive effect,” big data tax enforcement relies on highly centralized tax source information and intelligent information utilization and monitoring, further enhancing corporate tax-related information’s gold content. The “bridge role” of tax enforcement can not only effectively improve the information asymmetry between the government and enterprises, but also reduce the self-certification cost of the enterprise as a financier and the verification cost of the funder, breaking through the information barriers of both parties, and ease financing constraints (Cai et al., 2021), which is beneficial for enterprises to increase R&D investment. The continuous improvement of the standard level of tax enforcement can reduce the possibility of excessive law enforcement, thereby reducing the institutional transaction costs of enterprises (Sun et al., 2019). Strengthening tax enforcement can significantly narrow the tax burden gap between enterprises and improve the fairness of the tax environment (Li et al., 2020). The improvement of tax compliance will reduce the earnings management of tax avoidance motivation and increase the proportion of preferential tax policies, resulting in additional policy spillover effects (Xu, 2021). Hence, we propose the following hypothesis:

*H2(b):* The improvement of tax enforcement intensity has a positive impact on enterprise R&D investment.

*H3:* Tax enforcement shows a corresponding moderating effect on the relationship between tax incentives and R&D investment.

## Materials and Methods

### Sample Selection and Data Sources

The initial research sample of this paper included China’s Shanghai and Shenzhen A-share listed companies from 2010 to 2020. Considering the impact of data objectivity and data quality on the research results, the following screening steps were performed on the initial sample: (1) Eliminate the samples of listed companies in the financial and insurance industries; (2) Exclude enterprises with special treatment such as ST and ^*^ST with abnormal financial conditions during the observation period, to avoid the impact of major changes in the business environment; (3) Retain only samples without missing R&D investment information and important financial data for at least five consecutive years in the observation period, to ensure the continuity of research data. After the above processing, unbalanced panel data with 19,156 valid observations covering 10 years are finally obtained. The enterprise financial data and basic information used in the empirical test are from the China Stock Market & Accounting Research Database (CSMAR database). The annual China Statistical Yearbook collects the regional tax revenue and other macroeconomic data used to measure the intensity of tax enforcement. Furthermore, to reduce the influence of outliers, this study minorizes the upper and lower 1% quantiles on all continuous variables. All data processing in this paper was carried out by Stata 15.0 statistical analysis software.

### Regression Model

This paper first examines the policy effect of tax incentives on enterprise R&D investment, using the following model:


(1) $$\begin{array}{c} R\&D_{i,t}=\alpha_{0}+\alpha_{1}Taxp_{i,t}+\alpha_{2}Roa_{i,t}+\alpha_{3}Lev_{i,t}+\alpha_{4}Growth_{i,t}\\ +\alpha_{5}G5_{i,t}+\alpha_{6}Cashflow_{i,t}+\alpha_{7}Capital_{i,t}+\alpha_{8}Size_{i,t}\\ +\lambda_{industry}+\lambda_{year}+\xi_{i,t} \end{array}$$

*R&D* is the explained variable, that is, the level of enterprise R&D investment, *Taxp* is the core explanatory variable, representing the tax benefits enjoyed by the enterprise, and the rest are a series of control variables. *λ_industry_* and *λ_year_* represent the fixed effects of industry and year, respectively.

This paper constructs model (2) to test the competitive hypotheses *H2(a)* and *H2(b)*.


(2) $$\begin{array}{c} R\&D_{i,t}=\beta_{0}+\beta_{1}Taxenforce_{i,t}+\beta_{2}Roa_{i,t}+\beta_{3}Lev_{i,t}\\ +\beta_{4}Growth_{i,t}+\beta_{5}G5_{i,t}+\beta_{6}Cashflow_{i,t}\\ +\beta_{7}Capital_{i,t}+\beta_{8}Size_{i,t}+\lambda_{industry}+\lambda_{year}+\xi_{i,t} \end{array}$$

*Tax enforce* represents the intensity of tax enforcement, and the meanings of other variables are as described above.

Based on model (1), the interaction term *Taxp×Taxenforce* is introduced, and model (3) is constructed to examine whether tax enforcement moderates the relationship between tax incentives and enterprise R&D investment:


(3) $$\begin{array}{c} R\&D_{i,t}=\gamma_{0}+\gamma_{1}Taxp_{i,t}+\gamma_{2}Taxp_{i,t}\times Taxenforce_{i,t}\\ +\gamma_{3}Taxenforce_{i,t}+\gamma_{4}Roa_{i,t}+\gamma_{5}Lev_{i,t}+\gamma_{6}Growth_{i,t}\\ +\gamma_{7}G5_{i,t}+\gamma_{8}Cashflow_{i,t}+\gamma_{9}Capital_{i,t}+\gamma_{10}Age_{i,t}\\ +\lambda_{industry}+\lambda_{year}+\xi_{i,t} \end{array}$$

### Variable Selection and Measurement

#### Explained Variable: R&D Investment (*R&D*)

Considering that the amount of R&D investment disclosed in annual reports is less comparable between different industries and enterprises of different scales, this paper selects the intensity of R&D investment as its measurement.

#### Explanatory Variable: Tax Incentives (*Taxp*)

Among China’s innovative tax preferential policy tools, the value-added tax preference has a narrow scope of application and is not directly related to corporate R&D activities (Shui et al., 2015). The enterprise income tax has a higher degree of preference and frequency of use. Therefore, based on the research of Shui et al. (2015), this paper uses the effective tax rate adjusted by deferred income tax as a proxy variable to measure the tax incentives, and comprehensively reflects the tax burden level after considering various income tax incentives. The lower the effective tax rate, the more tax benefits it enjoys.

#### Explanatory Variable: Intensity of Tax Enforcement (*Taxenforce*)

Drawing on the research of Mertens (2003) and Chen et al. (2016), the expected tax revenue of various regions in China is estimated through regression model (4) and then compared with the actual tax revenue of the current year to measure the intensity of tax enforcement in different regions:


(4) $$\begin{array}{c} \frac{Tax_{i,t}}{GDP_{i,t}}=\sigma_{0}+\sigma_{1}\frac{Industy1_{i,t}}{GDP_{i,t}}+\sigma_{2}\frac{Industy2_{i,t}}{GDP_{i,t}}+\\ \sigma_{3}\frac{Industy3_{i,t}}{GDP_{i,t}}+\sigma_{4}\frac{Ex_{i,t}}{GDP_{i,t}}+\xi_{i,t}\; \end{array}$$

*Tax* is the actual tax revenue of each province at the end of the year, *GDP* is the gross domestic product of each province, *Industry1, Industry2,* and *Industry3* refer to the total output value of the primary, secondary, and tertiary industries, respectively, and *Ex* indicates the total import and export value of each province. The above variables were substituted into the model to obtain the estimated correlation coefficient, and then the expected value of $Tax_{i,t}$ */* $GDP_{i,t}$ was calculated. This paper uses the ratio of the actual value to the expected value to measure the tax enforcement intensity of each province. The higher the value, the stronger the tax enforcement in the area.

#### Control Variables

The decision of enterprises to carry out innovation activities will be influenced by their characteristics and the industrial environment. This paper controls the observable enterprise characteristic factors that affect the level of R&D investment from multiple dimensions and adds the ratio of an asset (*Roa*), financial leverage (*Lev*), enterprise growth (*Growth*), equity concentration (*G5*), cash flow ratio (*Cashflow*), tangible asset ratio (*Capital*) and enterprise size (*Size*) into the regression model. In addition, this paper sets dummy variables of year effect (*year*) and industry characteristic (*industry*) to smooth the effects of time factors and industry differences on innovation activities. The specific interpretation and measurement method of each variable are shown in Table 1.

**Table 1:** Variable names and identification.

**Types**	**Identification**	**Name**	**Measurement method**
Dependent variable	R&D investment	*R&D*	R&D investment/operating income
Independent variables	Tax incentives	*Taxp*	(income tax expense—deferred income tax expense)/earnings before interest and tax
	Intensity of tax enforcement	*Taxenforce*	The ratio of the actual value of the dependent variable to the predicted value in model (4)
Control variables	Ratio of asset	*Roa*	Net profit/average balance of total assets
	Financial leverage	*Lev*	Liabilities/total assets
	Enterprise growth	*Growth*	Business revenue growth rate
	Equity concentration	*G5*	The sum of the squares of the shares held by the top five shareholders
	Cash flow ratio	*Cashflow*	Cash flow of operating and investing activities/total assets
	Tangible asset ratio	*Capital*	Net fixed assets at the end of the year/total assets at the end of the year
	Enterprise size	*Size*	The logarithm of the total assets of the enterprise
	Time effect	*Year*	Virtual variable
	Industry effect	*Industry*	Virtual variable, set up according to the 2012 classification standards of the China Securities Regulatory Commission

## Results and Analysis

### Descriptive Statistics

Table 2 lists the descriptive statistics for the main variables. The average *R&D* index, which measures the intensity of R&D investment, is 4.70%. Compared with the R&D investment of enterprises in innovative countries, which is usually no less than 5% of operating revenue, the overall R&D investment level of Chinese listed companies is still relatively low (Lin et al., 2013). The gap between the maximum and minimum values of this indicator is large, which reflects the non-equilibrium state of R&D investment among enterprises. The average value of *Taxp* is 16.50%, and the median is 15.60%, indicating that most listed companies enjoy different income tax incentives. The average value of *Taxenforce* is 1.0007, and the minimum and maximum values are 0.5825 and 1.7457, respectively, indicating that the actual tax revenue of each region is generally slightly higher than the expected tax revenue, and the tax enforcement intensity of different regions is quite different.

**Table 2:** Descriptive statistics.

**Variable**	**Obs**	**Mean**	**SD**	**Med**	**Min**	**Max**
*R&D*	19,156	0.0470	0.0520	0.0361	0.0000	1.3745
*Taxp*	19,156	0.0374	0.2043	0.0701	−0.5102	0.4125
*Taxenforce*	19,156	1.0007	0.2587	0.9490	0.5825	1.7457
*Roa*	19,156	0.0429	0.0820	0.0409	−1.2759	4.4890
*Lev*	19,156	0.4012	0.2041	0.3887	0.0075	4.9952
*Growth*	19,156	0.1341	0.2389	0.1045	−0.2541	0.7144
*G5*	19,156	0.1563	0.1114	0.1288	0.0013	0.7942
*Cashflow*	19,156	−0.0149	0.1068	−0.0064	−3.3034	1.5137
*Capital*	19,156	0.2114	0.1459	0.1840	0.0000	0.8758
*Size*	19,156	22.1276	1.2912	21.9451	17.8061	28.6365

### Tax Incentives and Enterprise R&D Investment

This study uses panel fixed effects to verify the relationship between tax incentives and R&D investment (Models 1–3 in Table 3). *Taxp_t_* has a regression coefficient of −0.0090 and is significant at the 1% level. Considering that the release of preferential tax policy effects may have a certain time lag, this paper extends the time window and introduces the tax incentives with lag 1 (*Taxp_t-1_*) and lag 2 (*Taxp_t-2_*) into the model above as explanatory variables. Columns (2) and (3) report that the regression coefficients of *Taxp_t-1_* and *Taxp_t-2_* are −0.0101 and − 0.0073, respectively, and both pass the 1% significance test, indicating that the tax incentives can encourage enterprises to increase capital investment in R&D activities. The policy effect is sustainable, providing supporting evidence for the “promotion theory,” but there is still room for improvement in incentive degree. Tax incentives have advantages in market intervention, management cost, and flexibility, and their impact on enterprise nature and industry choice is neutral. Enterprise R&D needs long-term financial support. Tax preference is a policy tool that can significantly improve the intensity of R&D investment, and continuous and stable tax incentives help enterprises to form good policy expectations and ensure the continuity of R&D investment. The above results show that the effect of current tax incentives is usually maximized in the next phase of R&D investment. The long cycle of R&D activities will exacerbate the lagging effect of preferential policies. Given this, the following tax preference proxy variables use the data with a lag of 1 period.

**Table 3:** Tax incentives and enterprise R&D investment.

Variables	*R&D*	*R&D*	*R&D*
	(1)	(2)	(3)
*Taxp_t_*	−0.0090^***^ (−5.6701)		
*Taxp _t-1_*		−0.0101^***^ (−5.8533)	
*Taxp _t-2_*			−0.0073^***^ (−3.7894)
*Roa*	−0.0371^***^ (−8.3791)	−0.0337^***^ (−7.3641)	−0.0350^***^ (−7.2543)
*Lev*	−0.0530^***^ (−25.8786)	−0.0519^***^ (−23.8641)	−0.0503^***^ (−21.3642)
*Growth*	−0.0092^***^ (−6.5001)	−0.0105^***^ (−7.0108)	−0.0115^***^ (−7.0692)
*G5*	−0.0220^***^ (−7.2041)	−0.0218^***^ (−6.6197)	−0.0218^***^ (−5.9780)
*Cashflow*	−0.0195^***^ (−6.3282)	−0.0222^***^ (−6.6960)	−0.0249^***^ (−6.7807)
*Capital*	−0.0148^***^ (−5.4275)	−0.0160^***^ (−5.4747)	−0.0156^***^ (−4.9092)
*Size*	−0.0003 (−0.9310)	−0.0004 (−1.2012)	−0.0005 (−1.2931)
*Constant*	0.0690^***^ (8.6982)	0.0777^***^ (9.2550)	0.0837^***^ (9.2026)
*Year effects*	Yes	Yes	Yes
*Industry effects*	Yes	Yes	Yes
N	18,946	16,650	14,348
Adj. *R*^2^	0.3215	0.3293	0.3222

*Robust standard errors in parentheses*.

*** *p* < 0.01.

### Tax Enforcement and Enterprise R&D Investment

Based on model (2), the impact of tax enforcement on enterprise R&D investment is investigated (Models 1 in Table 4). The regression coefficient of *Taxenforce* is 0.0052, and it is significant at the level of 1%, which means that the promotion effect of “governance effect” and “incentive effect” caused by tax enforcement on enterprise innovation activities may exceed the negative impact brought by its “taxation effect” and “rent-seeking effect.” To avoid the loss of tax revenue, the law endows the tax authorities with the power of tax inspection and disposal, which gives them have stronger supervision ability than other shareholders. In addition, they have fewer “free-riding” considerations, thus showing a strong willingness to supervise. As a potential external governance mechanism, tax enforcement can effectively exert its governance and incentive effects, restrain the opportunistic behavior of controlling shareholders and management, such as related party transactions, insider trading, and on-the-job consumption, alleviate the moral hazard and adverse selection caused by information asymmetry, and reduce the direct and indirect agency costs derived therefrom. It can assist stakeholders to effectively supervise enterprise resource allocation decision-making, and weaken the negative effect of the actual tax burden on the capitalization rate of R&D expenditure. At the same time, mitigating the conflict of interest between the principal and the agent of the enterprise will help to enhance the consistency of their value maximization goals, improve the operation and investment efficiency, and guide the allocation of resources to the field of R&D and innovation.

**Table 4:** Tax enforcement and enterprise R&D investment and its moderating effect.

Variable	*R&D*	*R&D*
	(1)	(2)
*Taxenforce*	0.0052^***^ (3.9938)	0.0059^***^ (4.3032)
*Taxp _t-1_*		−0.0141^**^ (−2.1703)
*Taxp _t-1_ × Taxenforce*		0.0037 (0.5924)
*Control, Year, Industry*	Yes	Yes
N	18,987	16,650
Adj. *R*^2^	0.3199	0.3300

*Robust standard errors in parentheses*.

*** *p* < 0.01;

** *p* < 0.05.

The implicit right of the tax authorities to have discretionary power objectively affects the tax preferences enterprises enjoy. Based on model (3), this paper examines whether tax enforcement moderates the promotion of tax incentives and enterprise R&D investment (Model 2 in Table 4). The regression coefficient of the interaction term *Taxp×Taxenforce* is 0.0037. Still, it has not passed the significance test. The interaction between the tax effect, rent-seeking effect, governance effect, and incentive effect of tax enforcement will be affected by the internal characteristics of enterprises and show heterogeneity. In this paper, the research samples are grouped according to the nature of property rights, and binary dummy variables are set to divide the enterprise attributes. The state-owned enterprises are assigned one, and the non-state-owned enterprises are assigned zero. The specific results are shown in Table 5.

**Table 5:** Group regression results of the adjustment effect of tax enforcement.

Variable	*R&D*	*R&D*
	*State = 1*	*State = 0*
	(1)	(2)
*Taxp_t-1_*	0.0049 (0.5079)	−0.0307^***^ (−3.5028)
*Taxp_t-1_ × Taxenforce*	−0.0104 (−1.1557)	0.0168^**^ (1.9652)
*Taxenforce*	−0.0011 (−0.4918)	0.0100^***^ (5.6276)
*Control, year, industry*	Yes	Yes
N	5,256	11,394
Adj. *R*^2^	0.2892	0.3319

*Robust standard errors in parentheses*.

*** *p* < 0.01;

** *p* < 0.05.

The regression coefficient of *Taxp×Taxenforce* is negative and insignificant in column (1), and 0.0168 in column (2), which passes the 5% significance test. This shows that for non-state-owned enterprises, the strengthening of tax enforcement will weaken the promotion effect of tax incentives on enterprise R&D investment, which has not been reflected in state-owned enterprises. In the process of tax preference recognition or approval, there are certain soft conditions as the standard. The tax department has some degree of flexibility in determining the tax object, tax calculation basis, tax reduction, exemption, etc., so it can flexibly adjust the tax enforcement intensity. State-owned enterprises have natural advantages in resource acquisition and market share, and special political connections allow them to enjoy “soft budget constraints.” At the same time, paying more taxes is an important way for state-owned enterprises to undertake policy burdens such as ensuring fiscal revenue. The attributes of public property rights determine their weak incentives for tax avoidance, and the lower tax cost sensitivity may have less impact on the effect of preferential tax policies caused by the change in tax enforcement intensity. In contrast, non-state-owned enterprises have stronger tax avoidance motives and aggressive tax avoidance methods. Although strong tax enforcement can effectively restrain enterprises from abusing tax incentives (Hoopes et al., 2012), tax enforcement that is too strict will also make it difficult for enterprises to enjoy preferential policies such as R&D investment plus deduction, increase their tax compliance cost, and reflect the “taxation effect” and “rent-seeking effect” of tax enforcement, which hurts the policy effect aimed at promoting enterprises to increase R&D investment (Li, 2018).

## Conclusion

This paper uses the data of Chinese A-share listed companies from 2011 to 2020 to conduct empirical research to test the policy effect of tax incentives on enterprise R&D investment, and examine the impact of tax enforcement on enterprise R&D behavior and its moderating effect on the relationship between tax incentives and enterprise R&D investment. The results reveal that China’s preferential tax policies positively affect enterprise innovation activities, and stable and continuous tax incentives can stimulate enterprises to increase R&D investment. Increasing the intensity of tax enforcement has a significant positive impact on enterprise R&D investment. However, the promotion effect of tax incentives is weakened, which is only reflected in non-state-owned enterprises.

The policy implications of the research conclusions of this paper are as follows: First, the collocation of tax types and preferential methods should be further optimized. The empirical results show that although preferential tax policies have played an expected incentive role in general, there are still opportunities for wider use of policy tools. From the perspective of policy design, as two representative tax incentives, the preferential tax rate of enterprise income tax and the addition and the additional deduction of R&D expenses, when superimposed, compared with enterprises enjoying the preferential tax rate, the enterprises applicable to the high tax rate can enjoy more pre-tax deductions, which dilutes the effect of tax incentives. Indirect preference focuses on the ex-ante incentives for enterprise innovation activities.

### Policy Implications

Compared with the direct tax rate preference, policymakers should further expand the application scope of indirect preference, learn from international practice, and enhance the accuracy of tax incentives by increasing the ratio of R&D expenses deduction, special fixed asset depreciation, etc. Second, enterprises should objectively view the impact of tax enforcement, face up to the role of tax enforcement as an external governance mechanism in promoting enterprise innovation activities, and cooperate with tax authorities in law enforcement with a positive attitude. Third, the tax department should further optimize the big data tax enforcement model, standardize the enforcement process and taxation services, fully release its incentive effect while improving the governance ability of enforcement behavior, create a fair, open, and transparent business environment, and effectively support and guide enterprises to carry out innovation activities.

## Data Availability Statement

Publicly available datasets were analyzed in this study. The study data were collected from the China Stock Market & Accounting Research (CSMAR). This data can be found at: https://cn.gtadata.com.

## Author Contributions

The author confirms being the sole contributor of this work and has approved it for publication.

## Funding

This research was funded by the National Social Science Foundation Youth Project “Research on Tax Incentive Policies Supported by Inclusive Enterprise Innovation” (17CGL045).

## Conflict of Interest

The author declares that the research was conducted in the absence of any commercial or financial relationships that could be construed as a potential conflict of interest.

## Publisher’s Note

All claims expressed in this article are solely those of the authors and do not necessarily represent those of their affiliated organizations, or those of the publisher, the editors and the reviewers. Any product that may be evaluated in this article, or claim that may be made by its manufacturer, is not guaranteed or endorsed by the publisher.
